# Mitochondrial Genome of the Eyeworm, *Thelazia callipaeda* (Nematoda: Spirurida), as the First Representative from the Family Thelaziidae

**DOI:** 10.1371/journal.pntd.0002029

**Published:** 2013-01-31

**Authors:** Guo-Hua Liu, Robin B. Gasser, Domenico Otranto, Min-Jun Xu, Ji-Long Shen, Namitha Mohandas, Dong-Hui Zhou, Xing-Quan Zhu

**Affiliations:** 1 State Key Laboratory of Veterinary Etiological Biology, Key Laboratory of Veterinary Parasitology of Gansu Province, Lanzhou Veterinary Research Institute, Chinese Academy of Agricultural Sciences, Lanzhou, Gansu Province, PR China; 2 College of Veterinary Medicine, Hunan Agricultural University, Changsha, Hunan Province, PR China; 3 Faculty of Veterinary Science, The University of Melbourne, Parkville, Victoria, Australia; 4 Dipartimento di Sanità Pubblica e Zootecnia, Università degli Studi di Bari, Valenzano, Bari, Italy; 5 Department of Pathogen Biology, Anhui Medical University, Hefei, Anhui Province, China; 6 College of Animal Science and Veterinary Medicine, Heilongjiang Bayi Agricultural University, Daqing, Heilongjiang Province, PR China; Washington University School of Medicine, United States of America

## Abstract

Human thelaziosis is an underestimated parasitic disease caused by *Thelazia* species (Spirurida: Thelaziidae). The oriental eyeworm, *Thelazia callipaeda*, infects a range of mammalian definitive hosts, including canids, felids and humans. Although this zoonotic parasite is of socio-economic significance in Asian countries, its genetics, epidemiology and biology are poorly understood. Mitochondrial (mt) DNA is known to provide useful genetic markers to underpin fundamental investigations, but no mt genome had been characterized for any members of the family Thelaziidae. In the present study, we sequenced and characterized the mt genome of *T. callipaeda*. This AT-rich (74.6%) mt genome (13,668 bp) is circular and contains 12 protein-coding genes, 22 transfer RNA genes and two ribosomal RNA genes, but lacks an *atp*8 gene. All protein-coding genes are transcribed in the same direction; the gene order is the same as those of *Dirofilaria immitis* and *Setaria digitata* (Onchocercidae), but distinct from *Dracunculus medinensis* (Dracunculidae) and *Heliconema longissimum* (Physalopteridae). Phylogenetic analyses of the concatenated amino acid sequence data for all 12 protein-coding genes by Bayesian inference (BI) showed that *T. callipaeda* (Thelaziidae) is related to the family Onchocercidae. This is the first mt genome of any member of the family Thelaziidae and should represent a new source of genetic markers for studying the epidemiology, ecology, population genetics and systematics of this parasite of humans and other mammals.

## Author Summary

Human thelaziosis is an underestimated parasitic disease caused by the eyeworm *Thelazia callipaeda* (Spirurida: Thelaziidae). Although this parasite is of significance in humans in many Asian countries, its genetics, epidemiology and biology are poorly understood. Mitochondrial (mt) DNA can provide useful genetic markers for fundamental investigations, but no mt genome had been characterized for any members of the family Thelaziidae. In this study, we sequenced and characterized the mt genome of *T. callipaeda*. This circular mt genome is 13,668 bp long and contains 12 protein-coding genes, 22 transfer RNA genes and two ribosomal RNA genes, but lacks an *atp*8 gene. Phylogenetic analyses of the concatenated amino acid sequence data for all 12 protein-coding genes by Bayesian inference showed that *T. callipaeda* is closely related to the family Onchocercidae, consistent with previous study. This is the first mt genome of any member of the family Thelaziidae, and represents a new source of genetic markers for studies of the epidemiology, ecology, population genetics and systematics of this parasite of human and animal health significance.

## Introduction

*Thelazia callipaeda* Railliet and Henry, 1910, known as the ‘oriental eye-worm’, because of its geographical distribution in Asian countries (including China, India, Japan, Korea and Thailand), is frequently reported as being responsible for thelaziosis of humans, carnivores (dogs, foxes and cats) and rabbits, causing mild to severe clinical signs (including lacrimation, epiphora, conjunctivitis, keratitis and/or sometimes corneal ulcers) [1]. Fortunately, thelaziosis can be treated effectively using anthelminthics, such as milbemycin oxime or macrocyclic lactones (e.g., moxidectin), and anti-inflammatory compounds [2]–[4]. Although *T. callipaeda* may seem to be of minor importance to some clinicians and scientists, human thelaziosis is highly endemic in some under-developed communities in Asia, particularly in China [3]. Clearly, scant attention has been paid to human thelaziosis, and there are difficulties in its clinical diagnosis and differentiation from allergic conjunctivitis, particularly when small numbers of adult or larval stages of *T. callipaeda* are present in the eyes of infected patients.

The transmission of human thelaziosis occurs when the intermediate host, a drosophilid fly of the genus *Phortica*, feeds on lacrimal secretions from humans and other animals, and ingests first-stage larvae (L1s) produced by adult females of *T. callipaeda*, which live together with males in the conjunctival sac. After being ingested by the fly, the *T. callipaeda* larvae migrate in the vector's body (i.e. testis of the male) and undergo development from the L1 to the infective, third-stage larvae (L3) within 14–21 days. Following this migration, the L3s of *Thelazia* emerge from the labella of the infected fly, are deposited on the eye, as the vector feeds on lacrimal secretions, and then develop into the dioecious adult stages in the ocular cavity within ∼35 days [5].

In spite of the significance of human thelaziasis, little is known about the biology and epidemiology of *T. callipaeda* and its close relatives [1], [3]. This relates mainly to a lack of reliable morphological characters for their specific identification and for comparative study. Although molecular tools, employing genetic markers in short regions of nuclear ribosomal and mitochondrial (mt) DNA, have found utility for taxonomic and epidemiological studies of some species, such as *T. gulosa*, *T. rhodesi*, *T. skrjabini*, *T. lacrymalis* and *T. callipaeda* [1], there is still a paucity of information on *T. callipaeda* in different human populations and countries around the world.

Mt genomes can provide markers for genetic and epidemiological investigations of spirurid nematodes (e.g., [6], [7]), and provide the potential to discover population variants or cryptic species and investigate transmission patterns linked to particular haplotypes [8]–[11]. In addition, mt proteomic datasets could be used for reassessing systematic relationships of *Thelazia* and other spirurids. Recent studies have shown that concatenated mt proteomic datasets can be used effectively to retest hypotheses regarding the systematic relationships of different groups of nematodes (e.g., [12], [13]). Such amino acid sequence datasets are relatively large and informative, usually achieving excellent phylogenetic signal and mostly attaining nodal support values of 98–100% in tree reconstructions [12], [13]. Long-range PCR-coupled sequencing and bioinformatic methods [14], [15] have underpinned these advances, which now allow mt proteomic barcodes to be defined rapidly for *Thelazia* spp. and their relatives from a range of mammalian and invertebrate hosts. Here, as a first step, we (i) determined the sequence and structure of the mt genome for *T. callipaeda*, (ii) assessed the phylogenetic position of this nematode in relation to other Spirurida for which whole mt sequence datasets are available, and (iii) discussed the implications of the new dataset as a new resource for future genetic studies of *T. callipaeda* populations.

## Materials and Methods

### Ethics statement

This study was approved by the Animal Ethics Committee of Lanzhou Veterinary Research Institute, Chinese Academy of Agricultural Sciences (Approval No. LVRIAEC2010-008). The dog from which the adult specimens of *T. callipaeda* were collected was handled in accordance with good animal practice (GAP) required by the Animal Ethics Procedures and Guidelines of the People's Republic of China.

### Parasites and total genomic DNA isolation

Adult specimens of *T. callipaeda* were collected from a conjunctival sac of an infected dog at a veterinary hospital in Zhanjiang, Guangdong Province, China. The worms were washed extensively in physiological saline, fixed in ethanol and then stored at −20°C until use. Upon thawing, the anterior and posterior ends of each nematode were cut off and cleared in lactophenol for subsequent morphological identification [16]. The mid-body section of each worm was used for the isolation of total genomic DNA by small-scale sodium dodecyl-sulphate (SDS)/proteinase K digestion [17] and mini-column purification (TIANamp Genomic DNA kit). The molecular identity of each specimen was then verified by PCR-based sequencing of regions in the *cox*1 and *rrn*S genes using an established method [18], [19], and both regions had 99% identity to previously published sequences for *T. callipaeda* from China and Italy (GenBank accession nos. AM042555 and AJ544858, respectively).

### Long-PCR, sequencing and annotation

**Figure 1:**
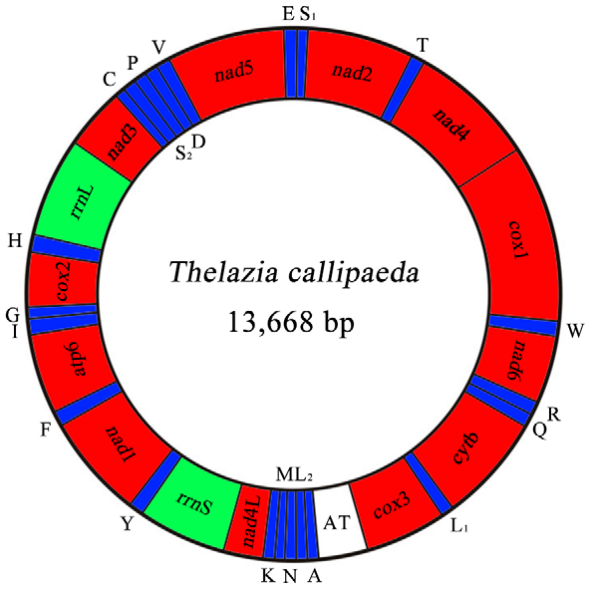
Organization of the mitochondrial genome of *Thelazia callipaeda*. Scale is approximate. All genes have standard nomenclature except for the 22 tRNA genes, which are designated by the one-letter code for the corresponding amino acid, with numerals differentiating each of the two leucine- and serine-specifying tRNAs (L_1_ and L_2_ for codon families CUN and UUR, respectively; S_1_ and S_2_ for codon families UCN and AGN, respectively). All genes are transcribed in the clockwise direction. ‘AT’ indicates the non-coding region.

Using primers (Table S1) designed to relatively conserved regions within the *cox*1 and *rrn*S regions (see Figure 1), the complete mt genome was amplified by long-PCR as two overlapping amplicons (∼5 kb and ∼9 kb) from the genomic DNA from the mid-body section of a single female specimen of *T. callipaeda*. PCR was conducted in 25 µl using 2 mM MgCl_2_, 0.2 mM each of dNTPs, 2.5 µl 10× Taq buffer, 2.5 µM of each primer and 0.5 µl LA *Taq* DNA polymerase (5 U/µl, Takara) in a thermocycler (Biometra) under the following conditions: 92°C for 2 min (initial denaturation), then 92°C for 10 s (denaturation), 58°C (5 kb) or 42°C (9 kb) for 30 s (annealing), and 60°C for 10 min (extension) for 10 cycles, followed by 92°C for 10 s, 58°C (∼5 kb) or 42°C (∼9 kb) for 30 s (annealing), and 60°C for 10 min for 20 cycles, with a cycle elongation of 10 s for each cycle and a final extension at 60°C for 10 min. Genomic DNA (80 ng in 2 µl) was added to PCR, and no-template and known-positive controls were included in each run. Amplicons were column-purified (Wizard PCR Preps, Promega). Subsequently, the amount of DNA in each purified amplicon was estimated spectrophotometrically (ND-1000 UV-VIS spectrophotometer, v.3.2.1, NanoDrop Technologies). Following an electrophoretic analysis of quality, purified amplicons were sequenced using a primer walking strategy [20]. The whole mt genome sequence (GenBank accession no. JX069968) was then assembled using the ContigExpress program of the Vector NTI software package v.6.0 (Invitrogen, Carlsbad, CA).

The mt genome was annotated using an approach similar to that of Yatawara et al. [21]. In brief, each protein-encoding mt gene was identified by local alignment comparison using amino acid sequences conceptually translated from corresponding genes from the mt genome of a reference species (i.e. *Setaria digitata*; accession number: NC_014282) [21]. The tRNA (*trn*) genes were identified using the program tRNAscan-SE [22] or by visual inspection [23]; rRNA (*rrn*) genes were predicted by comparison with those of *S. digitata* [21].

### Phylogenetic analysis of concatenated amino acid sequence data

The amino acid sequences conceptually translated from individual genes of the mt genome of *T. callipaeda* were concatenated. Selected for comparison were concatenated amino acid sequences predicted from published mt genomes from key nematodes representing the order Spirurida, including the superfamily Filarioidea (*Acanthocheilonema viteae* [24], *Brugia malayi* [25], *Chandlerella quiscali* [24], *Dirofilaria immitis* [26], *Loa loa* [24], *Onchocerca flexuosa* [24], *O. volvulus* [27], *S. digitata* [21] and *Wuchereria bancrofti* [7]), the superfamily Dracunculoidea (*Dracunculus medinensis*) and the superfamily Physalopteroidea (*Heliconema longissimum*) [13] (GenBank accession numbers NC_016197, NC_004298, NC_014486, NC_005305, NC_016199, NC_016172, AF015193, NC_014282, JN367461, NC_016019 and NC_016127, respectively), using *Ascaris suum* (GenBank accession number HQ704901) as an outgroup [28]. All amino acid sequences (considering all homologous characters) were aligned using MUSCLE [29] and then subjected to phylogenetic analysis using BI as described previously [30], [31]. Phylograms were drawn using the Tree View program v.1.65 [32].

## Results and Discussion

### General features of the mt genome of *T. callipaeda*

The complete mt genome sequence of *T. callipaeda* (GenBank accession no. JX069968) was 13,668 bp in length (Figure 1). This genome contains 12 protein-coding genes (*cox*1–3, *nad*1–6, *nad*4L, *atp*6 and *cyt*b), 22 *trn* genes, two *rrn* genes (*rrn*L and *rrn*S) and a non-coding (control or AT-rich) region, but lacks an *atp*8 gene (Table 1). The gene content and arrangement are the same as those of *D. immitis* and *S. digitata* [21], [26], but distinct from those of *D. medinensis* (rearrangement markedly) and *H. longissimum* (tRNA-Met and tRNA-Val change) [13]. All genes are transcribed in the same direction. In addition, the mt genes of *T. callipaeda* overlap by 102 nt in 14 locations (1 to 32 nt per location) (Table 1). The mt genome of *T. callipaeda* has 14 intergenic regions, which range from 1 to 62 nt in length. The longest region is between tRNA-Pro and tRNA-Asp (Table 1).

**Table 1:** The organization of the mitochondrial genome of *Thelazia callipaeda*.

Gene/Region	Positions	Size (bp)	Number of aa^a^	Ini/Ter codons	Anticodons	In
*cox*1	1–1647	1647	548	ATT/TAA		+7
tRNA-Trp (W)	1656–1710	55			TCA	+8
*nad*6	1743–2201	459	152	TTG/TAA		+32
tRNA-Arg (R)	2193–2258	66			ACG	−9
tRNA-Gln (Q)	2258–2310	53			TTG	−1
*cyt*b	2315–3397	1083	360	ATT/TAA		+4
tRNA-LeuCUN (L_1_)	3397–3452	55			TAG	−1
*cox*3	3450–4232	783	260	ATA/TAA		−3
Non-coding region	4233–4560	328				0
tRNA-Ala (A)	4561–4620	60			TGC	0
tRNA-LeuUUR (L_2_)	4624–4679	56			TAA	−1
tRNA-Asn (N)	4673–4733	61			GTT	−8
tRNA-Met (M)	4735–4793	59			CAT	+1
tRNA-Lys (K)	4794–4851	58			TTT	0
*nad*4L	4854–5090	237	78	TTG/TAA		+2
*rrn*S	5091–5756	666				0
tRNA-Tyr (Y)	5756–5812	57			GTA	−1
*nad*1	5813–6712	900	299	TTG/TAA		0
tRNA-Phe (F)	6681–6741	61			TTG	−32
*atp*6	6742–7323	582	193	ATT/TAG		0
tRNA-Ile (I)	7325–7381	57			GAT	+1
tRNA-Gly (G)	7383–7439	57			TCC	+1
*cox*2	7443–8147	705	234	ATA/TAG		+3
tRNA-His (H)	8138–8194	57			GTG	−10
*rrn*L	8194–9159	966				−1
*nad*3	9152–9487	336	111	TTG/TAG		−8
tRNA-Cys (C)	9488–9543	56			GCA	−1
tRNA-SerUCN (S_2_)	9543–9594	52			TGA	0
tRNA-Pro (P)	9596–9650	55			AGG	0
tRNA-Asp (D)	9712–9767	56			GTC	+62
tRNA-Val (V)	9769–9826	58			TAC	+11
*nad*5	9825–11417	1593	530	TTG/TAG		−1
tRNA-Glu (E)	11420–11475	56			TTC	+1
tRNA-SerAGN (S_1_)	11475–11526	52			TCT	0
*nad*2	11507–12367	861	286	TTG/TAA		−20
tRNA-Thr (T)	12375–12434	60			TGT	+6
*nad*4	12429–13661	1233	410	TTG/TAG		−5

a The inferred length of amino acid (aa) sequence of 12 protein-coding genes; Ini/Ter codons: initiation and termination codons.

In: Intergenic nucleotides.

The nucleotide content of the entire mt genome sequence of *T. callipaeda* is biased toward A+T (74.6%), in accordance with mt genomes of other nematodes of the order Spirurida (e.g., [21], [26]) (Table 2). One non-coding region (AT-loop) (328 bp), located between *cox*3 and tRNA-Ala, has the highest A+T content of 79.6% (Table 2). AT- and GC-skews of the whole mt genome were calculated for *T. callipaeda* and other spirurid nematodes studied to date (see Table 3). This composition of the mt genome sequence of *T. callipaeda* was strongly skewed away from A, in favour of T (AT skew = −0.40), and the GC skew was 0.449 (Table 3). All spirurid nematodes reported to date and in the present study show strand asymmetry (GC skew between 0.354 and 0.521) (Table 3).

**Table 2:** Comparison of A+T content (%) of the mitochondrial genomes of some spirurid nematodes.

Gene/region	AV	BM	CQ	DI	DM	HL	LL	OF	OV	SD	TC	WB
*atp*6	75.21	75.09	80.14	71.88	72.40	77.89	76.46	73.71	72.99	74.23	74.23	76.63
*cox*1	67.36	68.98	70.28	67.88	68.21	71.69	69.48	69.70	67.03	69.10	67.88	67.70
*cox*2	66.81	68.96	73.25	69.15	68.25	74.71	71.53	68.10	69.24	69.38	67.38	70.57
*cox*3	71.54	72.69	76.92	71.79	71.54	75.93	76.20	72.18	71.79	72.56	72.41	74.33
*cytb*	72.32	73.97	76.13	72.25	72.14	79.30	75.35	73.65	72.11	72.34	73.68	72.70
*nad*1	73.43	73.55	75.85	72.94	72.29	75.69	72.85	71.60	69.78	72.78	73.22	72.52
*nad*2	74.68	77.61	82.39	74.39	76.93	82.92	77.26	75.56	74.30	76.49	77.35	75.71
*nad*3	79.82	79.35	81.71	77.15	75.89	83.18	79.82	76.56	76.11	77.06	80.24	84.27
*nad*4	73.98	76.31	78.05	74.55	72.32	80.36	75.75	74.05	73.15	76.91	75.59	73.88
*nad*4L	76.89	82.08	83.33	77.37	74.39	82.05	81.09	77.73	78.60	76.76	80.17	80.66
*nad*5	71.93	74.81	78.17	73.75	73.64	78.93	74.03	73.62	72.87	74.81	73.82	74.69
*nad*6	77.19	81.46	82.89	80.57	76.26	81.74	81.98	81.11	79.11	82.44	80.17	80.04
*rrn*S	75.48	76.04	76.85	75.84	73.59	80.50	76.56	75.84	74.71	74.55	75.68	75.30
*rrn*L	77.78	80.78	80.25	79.55	76.70	81.81	78.65	77.71	76.95	79.40	77.43	79.01
AT-loop	83.37	85.11	86.49	85.91	74.75	96.75	83.68	79.93	85.32	86.36	79.57	83.71
Entire	73.54	75.46	77.67	74.16	72.72	79.11	75.54	74.17	73.30	75.14	74.57	74.59

Spirurid nematodes including *Thelazia callipaeda* were arranged in alphabetical order: AV: *Acanthocheilonema viteae*, BM: *Brugia malayi*, CQ: *Chandlerella quiscali*, DI: *Dirofilaria immitis*, DM: *Dracunculus medinensis*, HL: *Heliconema longissimum*, LL: *Loa loa*, OF: *Onchocerca flexuosa,* OV: *Onchocerca volvulus*, SD: *Setaria digitata*, TC: *Thelazia callipaeda*, WB: *Wuchereria bancrofti*, Entire: entire mt genome.

**Table 3:** Nucleotide composition of the mitochondrial genomes of spirurid nematodes, including that of *Thelazia callipaeda.*

Species	Nucleotide frequency (%)				Whole genome sequence		
	A	T	G	C	A+T%	AT skew	GC skew
*Acanthocheilonema viteae*	19.56	53.98	19.26	7.20	73.54	−0.468	0.456
*Brugia malayi*	21.60	53.86	16.82	7.72	75.46	−0.428	0.371
*Chandlerella quiscali*	23.02	54.65	15.92	6.41	77.67	−0.407	0.426
*Dirofilaria immitis*	19.26	54.90	19.28	6.56	74.16	−0.481	0.492
*Dracunculus medinensis*	20.12	52.60	20.75	6.53	72.72	−0.447	0.521
*Heliconema longissimum*	26.22	52.89	14.14	6.75	79.11	−0.337	0.354
*Loa loa*	20.78	54.76	17.73	6.73	75.54	−0.450	0.450
*Onchocerca flexuosa*	20.30	53.88	18.60	7.23	74.17	−0.430	0.440
*Onchocerca volvulus*	19.26	54.04	19.84	6.86	73.30	−0.474	0.486
*Setaria digitata*	19.42	55.71	18.14	6.72	75.14	−0.483	0.459
*Thelazia callipaeda*	22.39	52.18	18.42	7.01	74.57	−0.40	0.449
*Wuchereria bancrofti*	20.14	54.45	18.20	7.21	74.59	−0.460	0.433

### Protein-encoding genes

The boundaries between protein-coding genes of the mt genome of *T. callipaeda* were determined by aligning its sequence and by identifying translation initiation and termination codons with those of *H. longissimum* and *S. digitata* [13], [21]. In this mt genome, all protein-coding genes had ATT, ATA and TTG as their initiation codons, and TAA or TAG as their termination codon. Incomplete termination codons (T or TA) were not identified, which is inconsistent with studies of some other nematodes, including *Anisakis simplex* (*s. l.*), *A. suum*, *Caenorhabditis elegans*, *S. digitata*, *Toxocara* spp. and *Trichinella spiralis* [21], [33]–[36]. Codons composed of A and T were more frequently used in protein-coding genes, reflecting the high A+T content in the mt genome of *T. callipaeda*. The most frequently used amino acid was Phe (19.3%), followed by Leu (13.4%), Val (7.6%), Gly (7.1%) and IIe (6.2%) (Table 4).

**Table 4:** Codon usage of *Thelazia callipaeda* mitochondrial protein-coding genes.

Amino acid	Codon	Number	Frequency (%)	Amino acid	Codon	Number	Frequency (%)
Phe	TTT	655	18.86	Met	ATA	79	2.27
Phe	TTC	14	0.40	Met	ATG	68	1.95
Leu	TTA	251	7.22	Thr	ACT	83	2.39
Leu	TTG	214	6.16	Thr	ACC	0	0
Ser	TCT	122	3.51	Thr	ACA	4	1.11
Ser	TCC	4	0.11	Thr	ACG	3	0.08
Ser	TCA	6	0.17	Asn	AAT	89	2.56
Ser	TCG	2	0.05	Asn	AAC	5	0.14
Tyr	TAT	179	5.15	Lys	AAA	39	1.12
Tyr	TAC	7	0.20	Lys	AAG	46	1.32
Stop	TAA	7	0.20	Ser	AGT	97	2.79
Stop	TAG	5	0.14	Ser	AGC	7	0.20
Cys	TGT	92	2.64	Ser	AGA	32	0.92
Cys	TGC	5	0.14	Ser	AGG	49	1.41
Trp	TGA	48	1.38	Val	GTT	200	5.76
Trp	TGG	34	0.97	Val	GTC	6	0.17
Leu	CTT	27	0.77	Val	GTA	29	0.83
Leu	CTC	0	0	Val	GTG	30	0.86
Leu	CTA	8	0.23	Ala	GCT	62	1.78
Leu	CTG	3	0.08	Ala	GCC	6	0.17
Pro	CCT	50	1.44	Ala	GCA	10	0.28
Pro	CCC	9	0.25	Ala	GCG	10	0.28
Pro	CCA	8	0.23	Asp	GAT	64	1.84
Pro	CCG	9	0.25	Asp	GAC	5	0.14
His	CAT	53	1.52	Glu	GAA	37	1.06
His	CAC	1	0.02	Glu	GAG	36	1.03
Gln	CAA	18	0.51	Gly	GGT	126	3.62
Gln	CAG	33	0.95	Gly	GGC	12	0.34
Arg	CGT	32	0.92	Gly	GGA	39	1.12
Arg	CGC	2	0.05	Gly	GGG	70	2.01
Arg	CGA	6	0.17	IIe	ATT	212	6.10
Arg	CGG	10	0.28	IIe	ATC	4	0.11

The total number of codons is 3,473.

Stop = Stop codon.

### Other genes

In the mt genome of *T. callipaeda*, the *rrn*L was located between tRNA-His and *nad*3, and *rrn*S was between *nad*4L and tRNA-Tyr (Table 1). The sizes of the *rrn*L and *rrn*S genes of *T. callipaeda* were 966 bp and 666 bp, respectively (Table 1). The 22 *trn* genes ranged from 52 to 66 bp in size. The secondary structures predicted for the latter genes were similar to those of *S. digitata* [21].

### Substitution ratios

**Figure 2:**
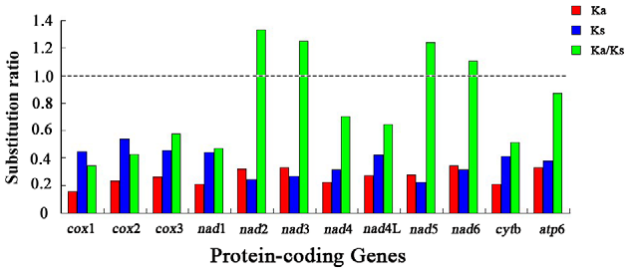
Substitution ratios in the mitochondrial genomes of spirurid nematodes. The rate of non-synonymous (Ka), the rate of synonymous (Ks) substitutions, and the respective ratios (Ka/Ks) for individual protein-coding genes are shown.

As synonymous and non-synonymous substitution rates assist in predicting evolutionary processes [37], the rate of non-synonymous substitutions (Ka), the rate of synonymous substitutions (Ks) and the Ka/Ks ratios were calculated for all 12 protein-coding genes encoded in the mt genomes of *T. callipaeda* and 11 other spirurid nematodes, including *A. viteae*, *B. malayi*, *C. quiscali*, *L. loa*, *S. digitata* and *W. bancrofti* (Table 3). The Ka/Ks ratio is a measure of selective pressures acting on gene that indicates neutral mutation (ka/ks = 1), negative or purifying selection (Ka/Ks of <1), and positive or diversifying selection (Ka/Ks of >1) [38], [39]. Here, *nad*2 showed the highest ratio, followed by *nad*3, while *cox*1 appeared to have the lowest ratio (Figure 2). Notably, the Ka/Ks ratio of eight protein-coding genes was <1 (range: 0.346 to 0.873), indicating that these genes are evolving under negative or purifying selection [40], [41]. The Ka/Ks ratio of 4 protein-coding genes (*nad*2, *nad*3, *nad*5 and *nad*6) was >1 (range: 1.105 to 1.331), suggesting that these genes have evolved under positive or diversifying selection [42].

### Sequence comparisons and phylogenetic relationships of *T. callipaeda* with selected members of the Spirurida

**Figure 3:**
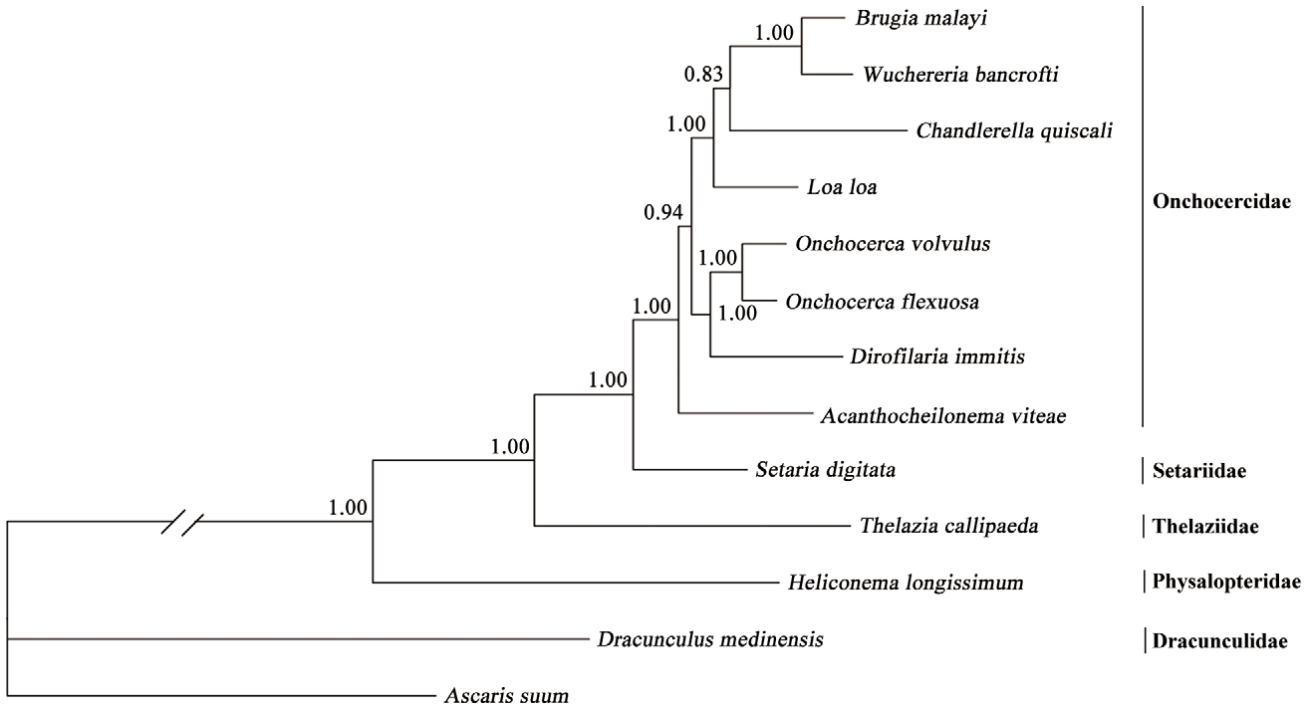
Relationship of *Thelazia callipaeda* with other selected spirurid nematodes based on mitochondrial sequence data. The concatenated amino acid sequences of 12 protein-coding genes were subjected to analysis by Bayesian inference (BI) using *Ascaris suum* as an outgroup. Posterior probability (pp) values are indicated.

The amino acid sequences predicted from individual protein-coding mt genes of *T. callipaeda* were compared with those of 11 other spirurid nematodes (see Table 5). Pairwise comparisons of the concatenated amino acid sequences revealed identities of 40.3–91.8% among them. Based on identity, COX1 was the most conserved protein, whereas *nad*4L and *nad*3 were the least conserved (see Table 5). Phylogenetic analysis of the concatenated amino acid sequence data for all 12 mt proteins showed that *T. callipaeda* (Thelaziidae) was a sister taxon to a clade containing *S. digitata* (Setariidae) and other members of the Onchocercidae, including *B. malayi* and *D. immitis*, consistent with results of a previous study [18]. Basal to these taxa were *H. longissimum* (Physalopteridae) and *D. medinensis* (Dracunculidae) (posterior probability = 1.00) (Figure 3).

**Table 5:** Differences (%) in mitochondrial amino acid sequences between *Thelazia callipaeda* and other nematodes.

Gene	AV	BM	CQ	DI	DM	HL	LL	OF	OV	SD	WB
*atp*6	55.4	58.0	49.2	58.1	18.8	52.6	57.5	58.5	57.0	57.0	57.5
*cox*1	80.8	83.2	80.1	84.3	52.1	70.1	83.6	83.8	84.1	83.8	83.8
*cox*2	64.7	65.9	66.4	67.7	40.4	48.5	63.8	69.8	67.2	69.0	65.7
*cox*3	69.1	68.3	64.5	65.6	36.5	54.7	67.2	68.3	68.7	66.4	69.5
*cyt*b	71.4	72.2	73.9	73.1	50.0	67.2	75.6	75.7	76.1	77.2	73.3
*nad*1	62.7	66.1	66.9	64.9	46.6	54.3	67.7	68.6	66.9	64.2	64.7
*nad*2	58.2	60.5	52.3	57.7	37.6	39.3	57.9	57.0	57.2	60.2	58.0
*nad*3	45.9	44.1	43.2	40.5	29.7	41.7	44.1	45.0	45.0	42.3	46.8
*nad*4	65.0	67.5	65.8	63.6	46.7	57.4	67.6	67.3	68.0	68.5	67.7
*nad*4L	25.6	25.6	25.6	25.6	20.5	23.4	28.2	24.4	23.1	26.9	26.9
*nad*5	66.6	65.8	63.8	65.8	38.7	54.8	64.0	65.3	66.0	64.3	67.0
*nad*6	49.0	48.7	47.0	50.0	29.0	35.2	45.0	47.7	45.0	52.3	49.3

Nematodes: AV: *Acanthocheilonema viteae*, BM: *Brugia malayi*, CQ: *Chandlerella quiscali*, DI: *Dirofilaria immitis*, DM: *Dracunculus medinensis*, HL: *Heliconema longissimum*, LL: *Loa loa*, SD: *Setaria digitata*, WB: *Wuchereria bancrofti*, OV: *Onchocerca volvulus*, OF: *Onchocerca flexuosa,* EmtG: entire mitochondrial genome.

### Fundamental and applied implications

Although much attention has been paid to soil-transmitted helminths as pathogens because of their major socioeconomic impact on human populations [43], [44], parasitic nematodes that cause relatively subtle, but chronic disease, such as members of the genus *Thelazia*, have been seriously neglected [3], [45]. The main reservoirs for human thelaziosis seem to be dogs, since they often live in areas populated by a large entomo-fauna [1], [46]. *T. callipaeda* is usually prevalent in dogs, cats and humans in disadvantaged, rural areas of the former Soviet Union [47] and the Asian continent, including China [48], India [49], Indonesia [50], Japan [51], Korea [52], Taiwan [53] and Thailand [54], [55]. More recently, *T. callipaeda* has also been reported in Europe, with a high prevalence (60%) in dogs being recorded in some areas of Southern Italy [56]. Autochthonous cases of canine thelaziosis have also been recorded in France [57], [58], Portugal [59], Spain [60] and Switzerland [61], suggesting that the latitude range of endemicity of canine thelaziosis in Europe (between 39° and 46°N) is similar to that of Asia (between 10° and 45°N for India and Japan) [56]. Interestingly, in spite of the high prevalence of canine thelaziosis reported for southern parts of Europe [56], only a small number of human cases have yet been reported in this geographical region [45].

In the present study, the characterization of the mt genome of *T. callipaeda* provides a foundation for the improved diagnosis of human thelaziosis using molecular methods as well as future, detailed studies of the population genetics and epidemiology/ecology of this parasite in Asia. As adult and larval stages of *T. callipaeda* from the eyes of patients cannot be identified reliably by morphology to species, molecular tools, using genetic markers in the first internal transcribed spacer (ITS-1) region of nuclear rDNA and *cox*1, have been used to support clinical diagnosis and to assist in undertaking molecular epidemiological investigations of *T. callipaeda* [1], [19]. Because sequence heterogeneity in ITS rDNA can be high in individual spirurid specimens (e.g., [62]), sometimes complicating sequence analyses, protein-coding mt genes appear to be better suited for such studies [19].

Having available the mt genome of *T. callipaeda* now sets the scene to develop combined DNA-based analytical and diagnostic tools, whereby mt genetic regions with differing levels of within-species divergence [19] might be used to explore haplotypic variation of individuals within and among *T. callipaeda* populations infecting humans and other definitive hosts as well as fly intermediate hosts, such as *Phortica variegata*. This could be done effectively using PCR-coupled mutation scanning and selective sequencing [17], already effectively applied, on a small scale, to *T. callipaeda* [19]. A previous investigation, employing *cox*1 alone, showed that, despite a relatively high degree of genetic variability among specimens isolated from Asia (i.e. China and Korea), no genetic variation was detected among individual specimens from different host species (i.e. dogs, cats and foxes) and localities within Europe (i.e. France, Germany, Italy, Netherlands, and Spain) [19]. These data were supported by additional studies [58]–[60], suggesting a genetically homogenized population in Europe, a tighter affiliation of this nematode to intermediate hosts than to the definitive hosts, and, thus, that the distribution of the parasite might be expected to resemble that of the vector [19]. Nonetheless, *cox*1 is a relatively conserved mt gene [63], and, to date, there is no genetic information for *T. callipaeda* from humans.

In the future, it would be interesting to assess whether various haplotypes or genotypes of *T. callipaeda* might relate to different clinical symptoms of thelaziosis in humans, and whether particular subpopulations of *T. callipaeda* undergo arrested development (hypobiosis, hibernation or aestivation) and survive for long periods of time in their intermediate hosts, as indicated in southern Europe [5]. Ecological aspects would be interesting to study in *T. callipaeda* in different countries and hosts, particularly dogs and cats, given the apparent complexity of the parasite's life cycle and biology. In addition, there is an applied human disease management imperative for improved molecular tools in being able to track the transmission of *T. callipaeda* and address the question as to whether infected domestic and wild canids and felids represent reservoir hosts for infection to humans in the same natural environment.

The characterization of the mt genome of *T. callipaeda* also stimulates a reassessment of the systematic relationships of nematodes within the order Spirurida using mt genomic/proteomic datasets. For decades, there have been controversies surrounding the systematics of members of the Spirurida (including superfamilies Acuarioidea, Aproctoidea, Diplotriaenoidea, Filarioidea, Gnathostomatoidea, Habronematoidea, Physalopteroidea, Rictularioidea and Spiruroidea) [64], [65]. Given the demonstrated utility of mt proteomic datasets, high phylogenetic signal and strong statistical support in trees [12], [13], there is now an opportunity to test the phylogenetic relationships of a wide range of spirurid nematodes using expanded mt datasets.

## Supporting Information

Table S1 **Sequences of oligonucleotide primers for amplifying regions of the mitochondrial genome of** ***Thelazia callipaeda.***
(DOCX)
