# Supplementary material for: Mitochondrial Genome of the Eyeworm, Thelazia callipaeda (Nematoda: Spirurida), as the First Representative from the Family Thelaziidae
Source: PLoS Negl Trop Dis. 2013 Jan 31;7(1):e2029. doi: 10.1371/journal.pntd.0002029 (PMC3561134; doi:10.1371/journal.pntd.0002029)
Supplement: Table S1 — Sequences of oligonucleotide primers for amplifying regions of the mitochondrial genome of Thelazia callipaeda. (DOCX) [file pntd.0002029.s001.docx]

**Table S1.** Sequences of oligonucleotide primers for amplifying regions of the mitochondrial genome of *Thelazia callipaeda.*

| **Primer designation** | **Sequence (5’ to 3’)^a^** | **Expected amplicon size** |
| --- | --- | --- |
| Short- PCR |  |  |
| NTF | TGATTGGTGGTTTTGGTA A | 590 bp |
| NTR | ATAAGTACGAGTATCAATATC |  |
| 12SF | GTTCCAGAATAATCGGCT A | 320 bp |
| 12SR | ATTGACGGATGRTTTGTACC |  |
| Long- PCR |  |  |
| TCCO1F | GATACTAAGAGGGGGGGTAGACCTTTATTGTT | 5 kb |
| TC12SR | CGGTTTAAATACAACTTTACTCCCGAACTA |  |
| TC12SF | TATTAGTTCGGGAGTAAAGTTGTAT | 9 kb |
| TCCO1R | AACAAATGCTGAAACAATAAAGGTCTAC |  |

^a^ R = A or G (according International Union of Pure and Applied Chemistry, IUPAC).
